# The value of natriuretic peptide testing for the diagnosis and prevention of heart failure in high-risk populations

**DOI:** 10.1515/almed-2024-0121

**Published:** 2024-08-19

**Authors:** Damien Gruson

**Affiliations:** Department of Laboratory Medicine, 70492 Cliniques Universitaires St-Luc and Université Catholique de Louvain, Brussels, Belgium; Pôle de recherche en Endocrinologie, Diabète et Nutrition, Institut de Recherche Expérimentale et Clinique, Cliniques Universitaires St-Luc and UCLouvain, Brussels, Belgium; Division on Emerging Technologies of the International Federation of Laboratory Medicine (IFCC), Milan, Italy

**Keywords:** heart failure, diabetes, hypertension, biomarker, prevention, natriuretic peptide

Heart failure (HF) represents a significant global health challenge, particularly in high-risk populations such as those with diabetes mellitus, hypertension, and cardiovascular diseases 1], [2], [3. Early diagnosis and prevention are crucial to mitigating the morbidity and mortality associated with HF [1, 4, 5]. Natriuretic peptides, specifically B-type natriuretic peptide (BNP) and its N-terminal fragment (NT-proBNP), have emerged as valuable biomarkers in the diagnosis and management of HF [3]. This editorial explores the value of natriuretic peptide testing in high-risk populations for the early diagnosis and prevention of heart failure.

Natriuretic peptides are hormones produced by the heart in response to ventricular volume expansion and pressure overload. BNP and NT-proBNP levels increase significantly in HF, reflecting the severity of the condition [3]. These biomarkers have been widely studied and validated for their diagnostic, prognostic, and therapeutic monitoring roles in HF.

Early diagnosis of HF is critical for initiating timely interventions that can improve patient outcomes [2, 6]. NT-proBNP testing is particularly valuable in high-risk populations. For example, in patients with diabetes mellitus, a condition that significantly increases the risk of developing HF, NT-proBNP testing can help identify subclinical HF. According to a study by Lapi et al., NT-proBNP testing in diabetic patients was associated with a higher propensity for early detection of HF [4]. The study showed that NT-proBNP testing was significantly more likely to be prescribed to patients with comorbid conditions such as ischemic cardiomyopathy, stroke, atrial fibrillation, and hypertension, all of which are risk factors for HF. Screening high-risk populations for HF using NT-proBNP can also aid in preventive strategies. Patel et al. demonstrated that NT-proBNP, when used alongside other screening tools like the WATCH-DM risk score, effectively identified individuals at high risk for HF [5]. The study found that selective NT-proBNP testing based on the WATCH-DM score efficiently pinpointed a high-risk primary prevention population with diabetes, who could benefit significantly from preventive therapies such as sodium-glucose cotransporter 2 (SGLT2) inhibitors.

The clinical utility of NT-proBNP extends beyond diagnosis. It provides prognostic information that can guide therapeutic decisions and monitor disease progression [3]. The cost-effectiveness of NT-proBNP testing has been established in various settings. For instance, the use of NT-proBNP in a two-step screening strategy with other biomarkers (e.g., high-sensitivity cardiac troponin) or imaging modalities (e.g., echocardiography) has been shown to capture a significant proportion of HF events while maintaining reasonable screening costs [1, 3].

**Figure 1:**
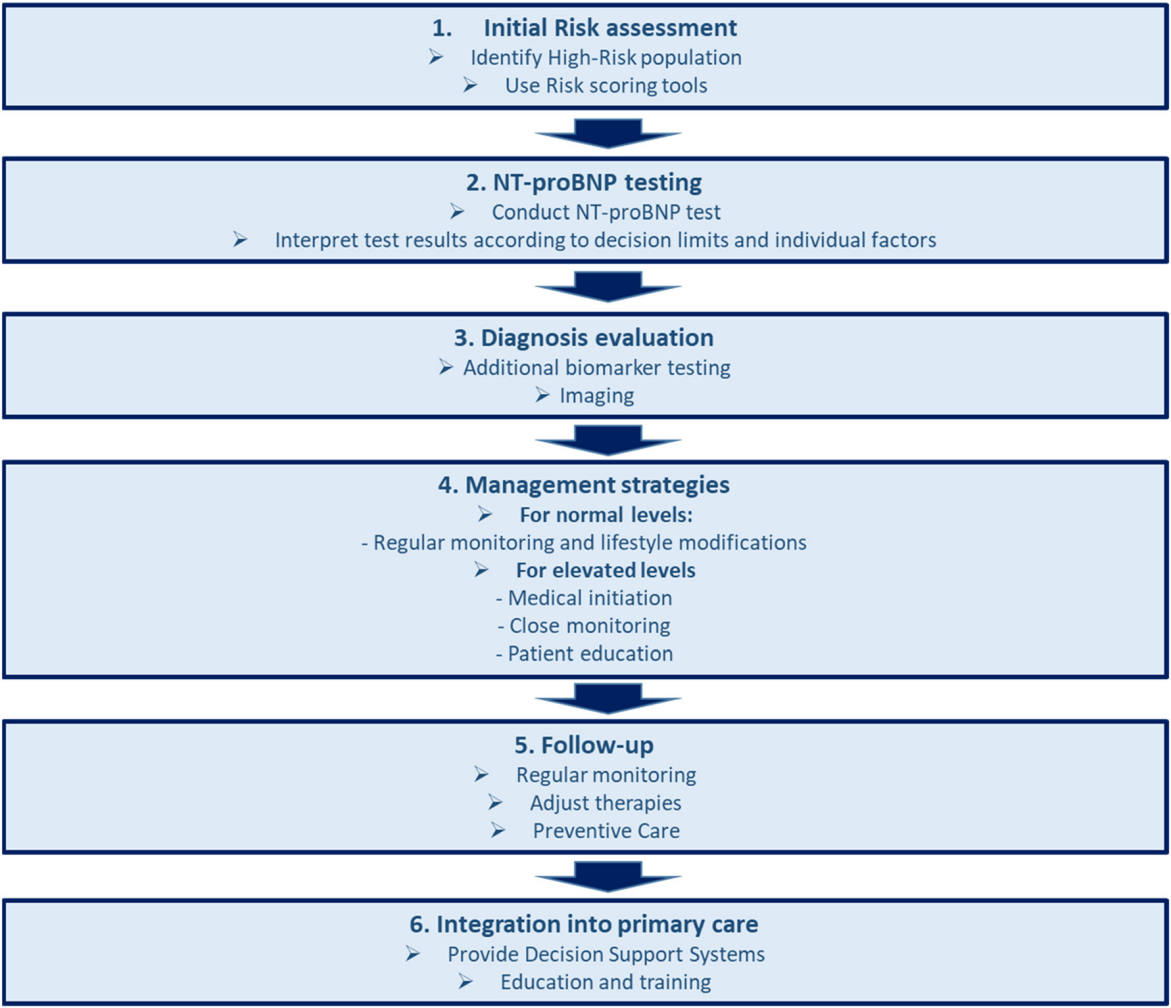
Screening and prevention of heart failure using NT-proBNP in high-risk populations. This flowchart illustrates the process of using NT-proBNP testing for screening and preventing heart failure in high-risk populations, including initial risk assessment, NT-proBNP testing, and subsequent management strategies based on test results.

The early identification of HF through natriuretic peptides testing allows for timely intervention with therapies that can slow disease progression, reduce hospitalizations, and improve survival. Medications such as SGLT2 inhibitors and angiotensin receptor-neprilysin inhibitors (ARNIs) have shown efficacy in reducing HF-related morbidity and mortality. Incorporating natriuretic peptides testing into routine clinical practice, especially in primary care settings, can thus enhance the overall management of high-risk patients [1]. Implementing natriuretic peptides testing in primary care settings can improve the management of high-risk populations. Lapi et al. highlighted the potential of NT-proBNP to support clinical decision-making in primary care, particularly in diabetic patients [4]. The study recommended the development of decision support systems to aid general practitioners in identifying patients who would benefit from NT-proBNP testing and subsequent HF management. Figure 1 illustrates a flowchart using NT-proBNP testing for early detection of HF in high-risk populations.

In conclusion, natriuretic peptide testing plays a critical role in the early diagnosis and prevention of heart failure in high-risk populations. Its use in clinical practice can lead to timely interventions, improved patient outcomes, and cost-effective healthcare. As evidence continues to support the integration of natriuretic peptides testing into routine screening and diagnostic protocols, healthcare providers should consider adopting these strategies to enhance the care of patients at risk for HF.
